# Successful Pregnancy Following Platelet-Rich Plasma Intraovarian Injection and In Vitro Maturation of Oocytes in a 47-Year-Old Woman: A Case Report

**DOI:** 10.7759/cureus.65281

**Published:** 2024-07-24

**Authors:** Nikos Petrogiannis, Kalliopi Chatzovoulou, Maria Filippa, Savvas Petrogiannis, Katerina Chatzimeletiou

**Affiliations:** 1 IVF Unit, Naval Hospital of Athens, Athens, GRC; 2 Unit for Human Reproduction, 1st Department of Obstetrics and Gynaecology, Aristotle University of Thessaloniki, Thessaloniki, GRC

**Keywords:** ovarian rejuvenation, blastocyst, oocyte, live birth, in vitro maturation

## Abstract

In vitro maturation (IVM) of oocytes represents an assisted reproductive technique that involves the minimal or absence of ovarian stimulation and is beneficial to specific groups of patients. It is based on the collection of immature cumulus-oocyte complexes (COCs) from antral follicles, which are cultured in vitro until they reach the metaphase II (MII) stage. Once maturation is completed, IVM oocytes are normally fertilized, as during a conventional IVF protocol. On the other hand, ovarian rejuvenation through the intraovarian platelet-rich plasma (PRP) injection represents an innovative procedure intended to restore ovarian fertility and development, and it is used to enhance ovarian stimulation outcomes. Here, we report a case of a 47-year-old woman who underwent an assisted reproductive technology cycle (ART) with PRP injection and IVM, which resulted in a successful pregnancy.

## Introduction

The ability to restore ovarian function safely is crucially important in assisted reproduction technology (ART) treatments. Ovarian platelet-rich plasma (PRP) injection, also known as ovarian rejuvenation, is a process whereby the ovaries are stimulated to induce follicle development and prompt good-quality oocytes for fertilization. Ovarian PRP involves the utilization of PRP, a concentrated solution of growth factors and cytokines derived from a patient’s blood [1]. More specifically, PRP is derived from whole blood, containing plasma (55%), red blood cells (41%), platelets, and white blood cells (4%), which are separated by centrifugation and into distinct components [2]. PRP is an autologous product, thus eliminating apprehensions regarding the risk of cross-contamination, disease dissemination, or immune reactions [3]. Once centrifuged and separated, red blood cells are removed, and the concentrated plasma is enriched in growth factors, such as transforming growth factor (TGF-β), vascular endothelial growth factor (VEGF), etc [4]. The group of components and growth factors present in PRP seem to be beneficial in enhancing collagen synthesis, macrophage activation, angiogenesis, and mitosis of endothelial cells and subsequently promote the recruitment of the best-candidate oocytes for fertilization, thus enhancing the fertility process [4]. To identify the success of the treatment, the patient is subjected to a series of analyses, where levels of AMH are expected to be increased while FSH and LH levels should be decreased. Another indication is determined through ultrasound examination to identify whether the antral follicle count (AFC) is increased as well.

On the other hand, another promising assisted reproductive technique for a specific group of patients with previous oocyte maturity insufficiency is the in vitro maturation (IVM) of oocytes. IVM involves the minimal or absence of ovarian stimulation and is beneficial to women with polycystic ovarian syndrome (PCOS) and/or patients who need a fertility preservation option before undergoing gonadotoxic treatment. IVM is based on the collection of immature cumulus-oocyte complexes (COCs) from antral follicles that are subsequently cultured in vitro until they reach the metaphase II (MII) stage [5-7]. Patients undergoing IVM receive no or minimal ovarian stimulation instead of a conventional controlled ovarian stimulation IVF (COS-IVF) protocol. Once maturation in the laboratory is completed, IVM oocytes are normally fertilized and treated exactly as the oocytes retrieved after conventional IVF [8]. IVM was basically designed as an alternative to standard ovarian stimulation protocols in order to overcome the negative effects and risks associated with hormonal stimulation of ovaries, such as ovarian hyperstimulation syndrome (OHSS) in high responders. IVM pregnancy rates and safety issues still remain controversial between IVF centers, making this subject a controversial one [9-12].

Here, we report a case of a 47-year-old woman who underwent an assisted reproductive technology cycle (ART) with PRP injection and IVM that resulted in a successful pregnancy.

## Case presentation

**Figure 1:**
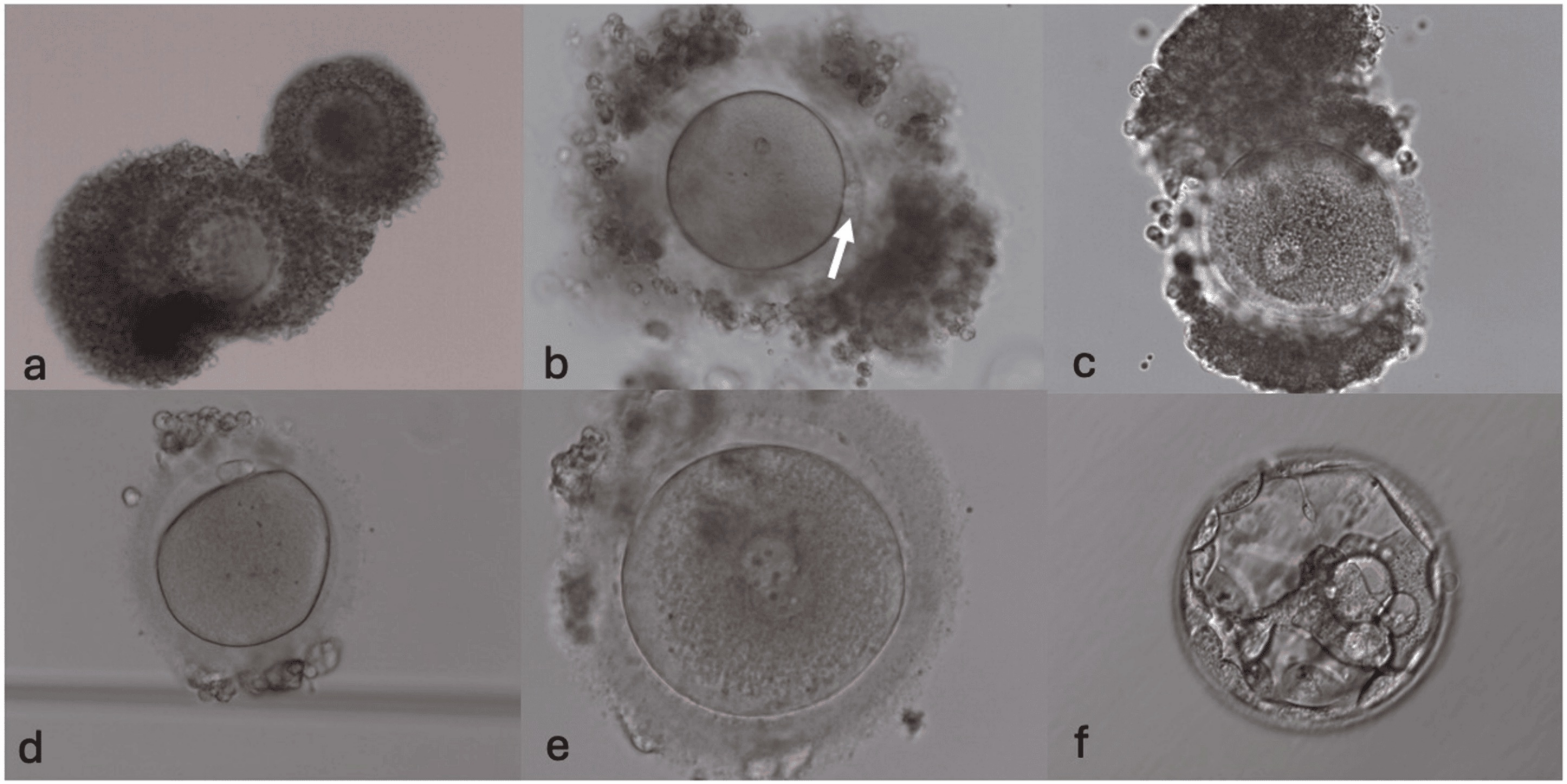
Maturation and development of the oocyte that was subjected to IVM and ICSI led to the blastocyst that was transferred to the uterus (a) The COCs at egg retrieval (day 0), showing signs of immaturity. (b) The first COC 23 hours post-IVM before denudation, showing a first polar body, indicated by a white arrow (mature MII oocyte). (c) The second COC 23 hours post-IVM being immature at the GV stage. (d) The oocyte after denudation, with the first polar body at a 12-hour position before ICSI. (e) Normal fertilization indicated by the presence of two pronuclei 16 hours post-ICSI. (f) Development of the fertilized oocyte to the blastocyst stage. IVM, in vitro maturation; ICSI, intracytoplasmic sperm injection; COC, cumulus-oocyte complex; MII, metaphase II; GV, germinal vesicle

A couple was referred to the Assisted Reproduction Unit of the Naval Hospital of Athens, Athens, Greece, for infertility treatment due to ovarian insufficiency. Previous IVF history of the patient included four cycles of conventional IVF protocol that didn’t result in a clinical pregnancy. After three years of diagnosed infertility, the 47-year-old female patient underwent ovarian PRP therapy, with her last menstrual cycle being completed one month before the PRP therapy. The patient has a normal cycle of 28 days, with a length of four to five days, while she is naturally menstruating. An ovarian ultrasound check was conducted 20 days post-PRP therapy, while menstruation started within 10 days after PRP. On day 2 of the menstrual cycle, the patient was subjected to a blood test examination to measure the levels of estradiol (E2) and progesterone, which were 71 pg/mL and 0.3 ng/mL, respectively. Then, the patient underwent ovarian stimulation with gonadotrophin (Puregon 100 IU for three days/in total 300 IU, European Medicines Agency) and clomiphene administration (Serpafar 50 mg for two days, Sanofi Aventis US, Bridgewater, NJ). The patient was monitored regularly by ultrasound and assessment of E2 levels. On day 6 of the menstrual cycle, three follicles were detected of diameters 10, 11, and 12 mm each. On day 7 of the menstrual cycle, transvaginal ultrasound-guided oocyte retrieval, by IVM needle type, was performed, and two COCs were retrieved by flushing ovarian follicles with FertiCult medium (FertiPro, Beernem, Belgium). The COCs were immature at oocyte retrieval (day 0), as expected, and they were subsequently cultured in LAG medium (Origio, Malov, Denmark) for three hours, followed by culture in IVM medium (Origio) for 22 hours in 6% CO_2_/7% O_2_ at 37°C (Figure 1a). Twenty-five hours post-IVM, COCs were denuded with the aid of hyaluronidase supplemented with serum albumin (Hyase-10X, Vitrolife Group, Gothenburg, Sweden), in order to assess the maturation state of oocytes prior to intracytoplasmic sperm injection (ICSI). Only one oocyte reached the MII stage, while the other one was immature at the germinal vesicle (GV) stage (Figures 1b, 1c). ICSI was performed with the husband’s sperm sample, which was produced and processed on day 1 post-retrieval (Figure 1d). Pronuclei (PN) check 16 hours post-ICSI revealed signs of normal fertilization, with the appearance of two PNs (Figure 1e). The zygote normally cleaved and reached the blastocyst stage on day 5 post-ICSI (Figure 1f). Embryonic quality was evaluated and categorized as a fair quality blastocyst (grade 5BC), according to Gardner’s scale. The blastocyst was then vitrified (VT601 Vitrification Media, Kitazato, Shizuoka, Japan) and transferred in the patient’s next cycle in a well-prepared endometrium, three hours post-thawing (VT602 Thawing Media, Kitazato).

Ten days after the embryo transfer, a positive serum β-hCG test was obtained (100 IU). Five days later, the β-hCG test was repeated (500 IU), and two days later, the β-hCG had reached 1100 IU. This pregnancy is still ongoing.

## Discussion

Intraovarian PRP reflects a breakthrough approach in ART treatments, aiming to restore ovarian function with very promising results. PRP is described as a plasma containing high concentrations of platelets, along with numerous growth factors, cytokines and chemokines, which play a pivotal role in ovarian tissue rejuvenation and repair. Their main roles are based on both cell proliferation and tissue growth, as well as the generation of an overall favorable environment, thus explaining the favorable effects of PRP on endometrial growth and receptivity via its anti-microbial and anti-inflammatory properties [13]. Data from metanalyses indicate that autologous intraovarian PRP infusion may restore ovarian function, promoting reactivation of the folliculogenesis process and enhancement of the hormonal profile. Subsequently, it may enable the achievement of a clinical pregnancy for a certain group of patients [14,15]. More specifically, intraovarian PRP injection was correlated to a statistically significant increase in serum anti-Müllerian hormone (AMH) levels [16], while also a post-treatment increase in the mean values of AFC was detected in the same study. Interestingly, post-PRP ICSI cycles were assessed for their success rates, indicating that all parameters (total number of retrieved oocytes, number of mature oocytes, number of two PN embryos, number of cleavage stage embryos, and cancellation rates) were better in the post-PRP ICSI group, compared to the controls [17]. As a result, PRP treatment may improve fertility parameters, especially in patients with diminished ovarian reserve. This novel and promising therapy has the potential to provide an alternative to these patients and lead to increased live birth rates. Although even if the available data are promising, there are still significant issues with their quality, and in general, there is a lack of data showing safety in the ART context. Therefore, intrauterine or intraovarian PRP is not recommended by the ESHRE-specific add-ons group outside strict research protocols in infertile women [18]. Moreover, results from a recent randomized controlled trial showed that the intraovarian PRP injection procedure does not improve mature oocyte yield or other parameters of IVF outcome in women less than 38 years old with an established IVF history of ovarian insufficiency [19]. Also, conclusions from a recent Cochrane Review were uncertain about the effect of intraovarian administration of PRP on outcomes of ART in infertile women [20]. Taken all together, the need for more well-designed randomized controlled trials is urgent in order to estimate its efficacy and identify potential biomarkers of ovarian reserve that will indicate the success of intra-ovarian PRP infusion.

On the other hand, IVM represents a procedure where minimal or absence of ovarian stimulation is needed in a specific group of patients. As such, it is supposed to be an advantageous technique compared to a conventional IVF protocol, as it requires less time, minimal medical monitoring, and fewer to no hormone injections and blood monitoring. While the effectiveness and safety of the technique still remain questionable [6,18], IVM itself is not considered an experimental/add-on technique and is indicated for a group of patients, as recently declared by the ESHRE Add-ons working group [18]. Overall, IVM is not recommended for infertile patients without specific indications (PCOS/high responders or fertility preservation) in the absence of long-term safety data, procedural reliability, and effectiveness [18].

## Conclusions

Here, we report a case of a 47-year-old patient who underwent an intraovarian PRP injection followed by an IVM cycle with minimal ovarian stimulation protocol. Out of the two COCs retrieved at egg collection and subjected to IVM culture medium, only one mature oocyte (MII) was obtained, which was successfully fertilized by ICSI. The developing zygote, which reached the blastocyst stage on day 5 post-ICSI, was vitrified and transferred following warming in the patient’s next cycle, resulting in a pregnancy that is currently ongoing. This case emphasizes the positive outcome of the use of intraovarian PRP injection, combined with an IVM protocol, in a patient with a poor ovarian profile and a low number of mature oocytes at oocyte retrieval. In the future, well-designed clinical trials will be mandatory to provide concrete evidence of any beneficial PRP treatments that may have an effect on ovarian rejuvenation and the achievement of a clinical pregnancy.
